# MicroRNAs in B cell development and malignancy

**DOI:** 10.1186/1756-8722-5-7

**Published:** 2012-03-08

**Authors:** Thilini R Fernando, Norma I Rodriguez-Malave, Dinesh S Rao

**Affiliations:** 1 Department of Pathology and Laboratory Medicine, UCLA, 10833 Le Conte Avenue, Los Angeles, CA 90095, USA; 2 Cellular and Molecular Pathology Ph.D. Program, Department of Pathology and Laboratory Medicine, UCLA, 10833 Le Conte Avenue, Los Angeles, CA 90095, USA; 3 Jonsson Comprehensive Cancer Center, UCLA, 650 Charles E. Young Drive South, Factor 8-684, Los Angeles, CA 90095, USA; 4 Broad Stem Cell Research Center, UCLA, 650 Charles E. Young Drive South, Factor 12-272, Los Angeles, CA 90095, USA; 5 Division of Biology, California Institute of Technology, 1200 E. California Blvd, Pasadena, CA 91106, USA

## Abstract

MicroRNAs are small RNA molecules that regulate gene expression and play critical roles in B cell development and malignancy. miRNA expression is important globally, as B cell specific knockouts of Dicer show profound defects in B cell development; and is also critical at the level of specific miRNAs. In this review, we discuss miRNAs that are involved in normal B cell development in the bone marrow and during B cell activation and terminal differentiation in the periphery. Next, we turn to miRNAs that are dysregulated during diseases of B cells, including malignant diseases and autoimmunity. Further study of miRNAs and their targets will lead to a better understanding of B cell development, and should also lead to the development of novel therapeutic strategies against B cell diseases.

## Introduction

In a relatively short time period, gene expression regulation by microRNAs (miRNAs) has changed the way that we view developmental and pathological processes. From initial discoveries in *C. elegans*, the identification of the novel small RNA biogenesis pathway and the identification of RNA interference, the field has moved rapidly [1-6]. The involvement of miRNAs in hematopoiesis has now been documented by numerous groups and they seem to regulate almost every aspect of hematopoietic development. In this review we focus on B cell development, where the importance of gene expression regulation has been appreciated for many years. miRNAs have emerged as critical regulators of gene expression and regulate many aspects of B cell development, and are dysregulated in B cell malignancies. Here, we review many of the studies that have been performed to delineate the roles of miRNAs in development and malignant transformation of B cells.

### MicroRNA biogenesis

miRNAs are non-protein coding RNAs of about 19-23 nucleotides. They are post-transcriptional gene regulators that bind to partially complementary sequences in the 3' UTR on target messenger RNA transcripts, thereby causing downregulation of the target [7]. They were first discovered in 1993 in *C. elegans *by Victor Ambros, during a study of *lin-14*. They identified a small RNA product encoded by *lin-4 *gene that is responsible for the downregulation of LIN-14 protein [2,3,8]. This central dogma of miRNA action has proven to stand the test of time, as miRNAs in most organisms are thought to behave similarly.

miRNAs can be grouped in to at least three categories depending on their genomic location: exonic miRNAs in non-coding genes, intronic miRNAs in non-coding genes and intronic miRNAs in protein-coding genes [9]. miRNAs are expressed as long primary RNA (pri-miRNA) as part of RNA polymerase II-driven transcript [10]. Therefore, it is possible that some miRNAs are co-regulated with their host gene as a part of transcriptional regulation during B cell development. The pri-miRNA is recognized by RNA binding protein DGCR8 and is processed by RNase III-type protein Drosha in the nucleus yielding a pre-mRNA [11,12]. Pre-miRNA is then exported to the cytoplasm by Exportin-5 where it is further processed by a second RNase III-type enzyme, Dicer, to produce a mature miRNA duplex [13]. The 19-25 nucleotide-long double stranded miRNA duplex is then unwound and incorporated into RNA-induced silencing complex (RISC), with strand selection based on thermodynamic properties. In the RISC, the miRNA binds to the target sequence in the 3' UTR via 6-8 nucleotide seed region and downregulates the expression of the target either by direct degradation or destabilization and eventually degradation of the target [14-16]. Since the repression is achieved by complementary base pairing via a relatively short seed sequence, miRNAs are predicted to have multiple targets. A genome wide statistical analysis has shown that one miRNA can have hundreds of targets, indicating their critical role in post translation regulation [17]. It should be noted that recently, a Dicer-independent miRNA biogenesis pathway has also been reported. This pathway utilizes the catalytic activity of Argonaute2 (Ago2) [18-21]. miR-451 is the best characterized miRNA that is produced independently of Dicer and is involved in erythropoiesis. The unusual short stem structure of pre miR-451 promotes the binding and processing by Ago2 [19].

miRNAs have already found to influence immune cell differentiation. Recently, it was found that Dicer and miRNA play vital roles in both early and late B cell differentiation [22,23]. Deletions of individual miRNA genes are associated with several immune defects. In many instances, dysregulated expression of miRNAs has been seen in malignancies in the immune system, which we will discuss in detail later in the review.

### B cell development

**Figure 1:**
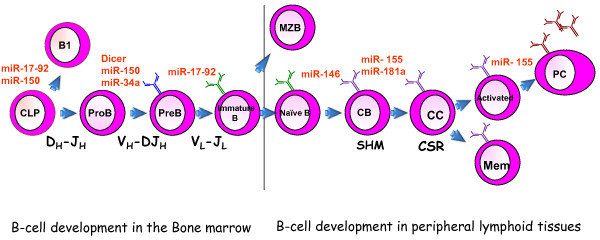
**miRNAs involved in B cell development in the bone marrow and periphery**. B cells development starts in the bone marrow and achieves a remarkable diversity of immunoglobulin loci by V(D)J recombination. Immature B cells migrate to the secondary lymphoid organs where they are activated by specific antigens. Once they are activated, they undergo proliferation and further differentiation into plasma cells that secrete antibodies or memory B cells that can be reactivated with a secondary infection. There are at least three different types of mature B cells: B1 cells, conventional follicular B2 cells and marginal-zone B cells. miRNAs that are involved in different stages of B cell development are indicated above the arrows. Secondary diversification of the immunoglobulin loci is achieved by SHM and CSR at the germinal center. Abbreviations, CLP: Common lymphoid progenitor; MZB: marginal zone B cells; CB: Centroblasts; CC: Centrocytes; PC: Plasma Cell; Mem: Memory cell; SHM: Somatic Hypermutation; CSR: Class switch recombination.

B cells are responsible for adaptive humoral immunity. B cell development is characterized by complex sequence of molecular events that is regulated by B - lineage transcription factors. It is evident that miRNAs play a major role in modulating the expression of these transcription factors and thereby the normal development of B cells. Conversely, dysregulation of miRNA expression is thought to be a key factor to the pathogenesis of B cell malignancies, including progenitor B cell-malignancies such as B-lymphoblastic leukemia (also referred to as B-Acute lymphoblastic leukemia or B-ALL) and mature B cell malignancies including several types of non-Hodgkin lymphoma. B cell development begins in the fetal liver and continues in the bone marrow of adult throughout the life (reviewed in [24,25]). The process of B cell formation starts in the bone marrow and ends in the peripheral secondary lymphoid organs such as the spleen (Figure 1). Here, we provide a primer on B cell development to orient the discussion on the role of miRNAs in B cells.

### Bone marrow B cell development

In the bone marrow, cellular stages of development include the common lymphoid progenitor, pro-B cell, pre-B cell and immature-B cell stages. These stages of B cells are defined by the expression and the re-arrangement of functional B cell receptor (BCR)/immunoglobulin (Ig) genes. Complex and elegant mechanisms have evolved to generate a diverse repertoire of BCRs against the vast variety of antigen that we encounter in our lifetime. This diversity is achieved by V (D)J recombination of the immunoglobulin locus, which is a process of somatic recombination that brings together various gene segments within the heavy and light chain loci. The heavy chain is assembled from Variable (V), Diversity (D) and Joining (J) gene exons that somatically recombine with the Constant (C) region exons to generate unique immunoglobulins of differing antigenic specificity. On the other hand, the light chain variable region is composed of only a V and J segments. The numbers of these gene segments are highly variable in different species and the different segments seem to have evolved from gene duplications from ancestral V-gene exons. Each V-, D-, and J- gene segment is flanked by DNA sequence called recombination signaling sequence, which is recognized by RAG1 and RAG2 enzymes, which mediate recombination. Random selection of these segments during V(D)J recombination and junctional diversity introduced by addition or subtractions of nucleotides at the junctions of these segments enable the production of vast variety of Ig (reviewed in [26-32]).

The stages of B cell development have been defined by the steps in V(D)J recombination. The Pro-B cell stage is characterized by rearrangement of Ig heavy chain, which occurs first. D-J joining occurs first, following which the DJ segment is joined to a V segment. D-J rearrangement starts in the common lymphoid progenitor and occurs mainly in early pro-B cells. V-DJ rearrangement occurs in late pro-B cells. The assembled heavy chain is then expressed on the surface of the pre-B cells along with a surrogate light chain. The pro-B cell to pre-B cell transition is accompanied by cell proliferation. Rearrangement of the light chain by V to J joining occurs during the pre-B cell stage. Successful assembly of light chain leads to the expression of complete IgM molecule at the surface and if the rearrangement is successful, signal transduction from IgM binding allows for differentiation into an immature B cell. Immature B cells that are not self-reactive leave the bone marrow as transitional B cells. Self-reactive immature B cells will either undergo apoptosis (clonal deletion), generate a new B cell receptor by receptor editing, or become unresponsive to antigen (reviewed in [26-29,31,33]).

Several transcription factors including PU.1, STAT5, E2A (E12 and E47), EBF, Pax-5, IKZFI and FOXP1 together with cytokines (IL-7, SCF) and chemokines(CXCL12), which are provided by the stromal cells, regulate the commitment and maintenance of B cells (reviewed in [34,35]). Absence of IL-7 signaling leads to developmental arrest at the pre-pro-B cell stage, showing the essential role of IL-7 signaling in B cell development in mice [36]. Downstream of IL-7, deletion of both STAT5 and STAT3 lead to developmental arrest at the pro-B stage [37,38]. Transcriptional regulation of B cell development is complex and involves the interplay of several transcription factors, including E2A, EBF, PAX5, FOXP1 and IKZF1. E2A-null mutant mice were unable to generate mature B cells [39], while Pax-5 is required for commitment to the B cell lineage [40]. Deletion of EBF blocked the pro-B to pre-B transition [41]. It has also been shown that Ebf^-/- ^hematopoietic cells do not express Pax-5 indicating that EBF acts upstream of Pax-5. This finding was further supported by the finding of an EBF-binding site in the Pax-5 promoter region [42]. Foxp1 is also an essential transcription factor for B cell development that is induced by E2A and in turn induces expression of the Rag enzymes. Deletion or knockdown of Foxp1 resulted in a reduction of B cell specific gene expression and interrupted the transition from pro-B cell to pre-B cell [35,43]. Other transcriptional regulators of B cell development include Ikzf1, which seems to play an important role in early lymphoid commitment.

These repeated cycles of DNA damage and repair may explain the reason for Pro-B and pre-B stages being more susceptible to oncogenic transformation. Also of interest, almost every transcriptional regulator of B cell development is disrupted in B-lineage malignancy. E2A has found in chromosomal translocations associated with B-ALL, and Pax-5 deletions are common in B-ALL [44-48]. Also in genome wide analysis, Pax-5 and EBF have been shown to be associated with B ALL [46]. Foxp1 translocation and overexpression is noted in mature B cell neoplasms, while IKZF1 is disrupted in pre-B-ALL [49-60]. Hence, the study of B cell development also informs the understanding of B cell malignancy pathogenesis.

### B cell development in the periphery

Mature B cells in the periphery are generally divided into B1 and B2 cells. B2 cells are conventional B cells that are derived from the bone marrow, undergo V(D)J recombination, and are part of the adaptive immune response (reviewed in [26]). A second set of recently described B cells, called B1 cells, are characterized by a limited immunoglobulin repertoire, and are part of the innate immune system (reviewed in [61]). These are best described in the mouse lymphoid system, where they express distinct sets of markers and are located mainly in the peritoneum and in the spleen. The lineage origin and relationship with conventional B2 cells is not entirely clear. The rest of our discussion will focus on B2 cells.

The majority of the cells in the spleen and in the circulation are thought to be B2 cells, and we focus the rest of the discussion in this section of such cells. B cells that have not encountered their specific antigen are called naïve B cells (CD 27-, IgD+). When a B cell binds to an antigen, it enters the germinal center of peripheral lymphoid tissues and eventually differentiates into plasma cells and memory cells. Plasma cells are a terminally differentiated, highly specialized B cell that secretes massive quantities of Ig, and whose differentiation is mediated by activation of plasma cell transcription factors such as Blimp1 and Xbp1 [62]. Memory B cells are long-lived and can be re-activated during a secondary infection. The entry of the activated B cells into primary lymphoid follicles results in the formation of germinal centers (GCs). Follicles with germinal centers have three distinct zones, namely dark, light and mantle zone. Rapidly dividing B cells which are called centroblasts form the dark zone of the GC. CBs eventually differentiate into non cycling centrocytes which make up the light zone of the GC. In the GC, secondary diversification of the immunoglobulin repertoire is achieved through somatic hypermutation and class switch recombination [63]. During this remarkable process, CBs can refine the specificity of their antigen receptor by somatic hypermutation of IgV gene, via the introduction of point mutations. If this process results in enhancement of the binding affinity for antigen, the B cell is selected for, and it survives to differentiate further. Also during the germinal center reaction, class switching from IgM to any other immunoglobulin can occur. Here, the variable portion of the heavy chain (VH exon) is brought adjacent to with different immunoglobulin constant regions (CH exons). This process allows making antibodies with different effector function. Remarkably, both of these processes-somatic hypermutation and class-switch recombination-are under the control of the same enzyme, adenosine-induced deaminase (AID) [64]. CC expressing Igs with enhanced affinity may eventually be released as memory B cells (CD 27+) from the GC. These memory cells are long lived and have potential to become antibody secreting cells during secondary infection (reviewed in [29,32,65-67]).

### MicroRNAs in bone marrow B cell development

As can be seen from the preceding discussion, bone marrow B cell development is carefully orchestrated, as only one gene locus is rearranged at a given time in a fixed sequence. Ig rearrangement is mediated by the sequential action of a gene regulatory network composed of transcription factors and growth factor receptors. miRNAs are known to act as post-transcriptional regulators of gene expression and it therefore stands to reason that they play a critical role in this network. The importance of miRNAs was first established by a seminal study that delineated a role for miRNAs in hematopoietic lineage choice selection. In this study, the authors determined that miR-181 (now known as miR-181a) was expressed most highly in B cells and that its overexpression in hematopoietic stem and progenitor cells led to increased output of B cells [68,69]. Further studies have shown additional miRNAs of importance in B cell development; in this section, we will focus on the role of miR-150, miR-34a and the miR-17-92 cluster in antigen independent B cell development at the bone marrow.

A general role for miRNAs in B cell development has also been established (Figure 1). The conditional knock-out of Dicer in early B cells led to a developmental arrest at the pro-B cell to pre-B cell transition, and also caused an effect on antibody diversification [70]. Gene-expression profiling from Dicer-deficient cells indicated that Bim, a known miR-17-92 cluster target, was upregulated in the mice. Functionally, B cell development was partially rescued by concurrent Bim ablation in Dicer-deficient mice. The implication of these studies was that miR-17-92, via repression of Bim, was the key player that was missing in Dicer-deficient B cell development. The miR-17-92 cluster is located on chromosome 13 and encodes six different miRNAs. The cluster is highly expressed in progenitor B cells and expression diminishes as cells mature. Ectopic expression of the cluster in mice resulted in expansion and activation of all lymphocyte populations in the periphery [71]. Compound heterozygous mutations of two target genes of miR-17-92, *Bim *and *Pten*, resulted in an accumulation of activated lymphocytes, indicating that partial repression of two targets may explain the majority of the miR-17-92-induced phenotype [71]. Taken together these data indicate that the miR-17-92 cluster plays a critical role in proliferation control in B cells, in B cell development and Ig rearrangement.

A second B cell-relevant miRNA, miR-150, is highly expressed in progenitor B cells and levels decrease at the pro-B cell to pre-B cell transition. miR-150 targets the c-Myb transcription factor in B cell development [72]. Confirming targeting, B cells that are deficient in miR-150 showed higher levels of c-Myb, while over-expression of miR-150 in transgenic mice caused reduced levels of c-Myb. Over-expression of miR-150 in mouse HSC led to a defect at the pro-B cell to pre-B cell transition [72]. Mice with targeted deletion of miR-150 had more B-1 cells in the spleen and peritoneal cavity and fewer B-2 cells, although they appeared phenotypically normal [73]. Knock-out mice for miR-150 at baseline contained higher serum concentrations of Ig classes, especially IgA, likely due to an expansion of B-1 cells [72]. Similarly, mice that were haploinsufficient for c-Myb had fewer mature B cells in the spleen and fewer B-1 cells, consistent with what was seen with the miR-150 transgenic mice [74] These data indicate a critical role for miR-150 during B cell development.

Along with miR-150, miR-34a is highly expressed in progenitor cells and downregulated and the pro-B cell to pre-B cell transition. Ig rearrangement has multiple check points dependent on TP53 [75]. TP53 targets miR-34a which in turn targets genes involved in cell cycle regulation, cell proliferation and apoptosis [76,77]. Among its targets are the anti-apoptotic protein, BCL2, and the transcription factor Foxp1 [43,78]. Mice with constitutive expression of miR-34a showed a block at the pro-B cell to pre-B cell transition with a reduction in mature B cells [43]. This arrest resulted from the inhibition of Foxp1 which is required for early B cell development. These findings elucidate a crucial role for miR-34a regulation at early B cell development.

### MicroRNAs in spleen and periphery B cell development

As in bone marrow B cell development, miRNAs as a whole, as well as specific miRNAs, have now been appreciated to play important roles in peripheral or antigen-dependent B cell development (Figure 1). At the global level, Dicer ablation in mature B cells (as opposed to early precursor B cells) using CD21-Cre resulted in an increase in marginal zone B cells and a decrease in follicular B cells [23]. Mice deficient for Dicer in mature B cells had an increased titer of autoimmune immunoglobulins with frank autoimmune disease in a proportion of the female mice. The mechanistic basis of these findings remains to be determined, but this study suggested that a miRNA may be responsible of regulating Bruton's tyrosine kinase. However, there are certainly some miRNAs that play major roles in B cell development; here we will focus on the role of miR-155, miR-146a and miR-181a in B cell development in the spleen and periphery.

In normal lymphopoiesis, miR-155 is expressed in moderate level in HSCs, at high levels in the germinal center and at much lower levels in mature B cells [79-81]. Expression of miR-155 is rapidly induced in B cells after engagement of the antigen receptor and exposure to inflammatory mediators [82,83]. Mice lacking miR-155 showed normal steady state immune cell populations; however, mice had a defective humoral response when immunized [84]. This response involved impaired germinal center formation and led to low antibody class switching to IgG in a B cell-intrinsic manner. The targets responsible for this appear to be multiple but likely include PU.1, SHIP1, and possibly AID [85-87]). The inhibition of the latter target is interesting, because the phenotype that is observed in the miR-155 deficient mice is one of decreased class-switching, whereas derepression of AID might be expected to cause increased class switching. The targeting of AID by miR-155 was extensively studied by mutating the binding site for miR-155 in the AID 3'UTR and these studies determined that disruption of the interaction did indeed lead to increased class-switching, and hence the overall effect of miR-155 likely includes additional targets [86,87]. Overall, these data are consistent with miR-155 playing an important role in regulating antigen-dependent B cell development. More recently, a second miRNA has been identified to regulate CSR- miR-181b overexpression in B cells was found to reduce the CSR rates, possibly by also downregulating AID [88].

The miR-146 family has distinct expression patterns amongst various hematopoietic lineages and is involved in maintaining lineage identity in lymphocytes. Vertebrates have two genomic copies of the miRNA, miR-146a and miR-146b, although the latter is likely a pseudogene [89]. miR-146a is induced by Toll-like receptor 4 and latent membrane protein 1 activation, and is NF-κB dependent [83,90]. miR-146a targets IRAK1 and TRAF6, two adapter proteins involved in Toll like receptor and interleukin 1 receptor signaling [91]. The role of miR-146a in B cells remains to be definitively determined, but overall B cell numbers are lower in miR-146a-deficient mice as the mice have a myeloproliferative disorder. Curiously however, many mice show dramatic follicular hyperplasia and active germinal centers with increased B cell function [92].

Some other miRNAs, including miR-125a, miR-125b, miR-99b and let-7e transcripts are preferentially expressed by the actively dividing centroblasts in germinal centers. In functional assays, miRNA-125b over-expression inhibited the differentiation of primary B cells [93]. Hence, it can be seen that several miRNAs show important roles during antigen-dependent B cell development.

### MicroRNAs in B cell lymphoma and leukemia

**Figure 2:**
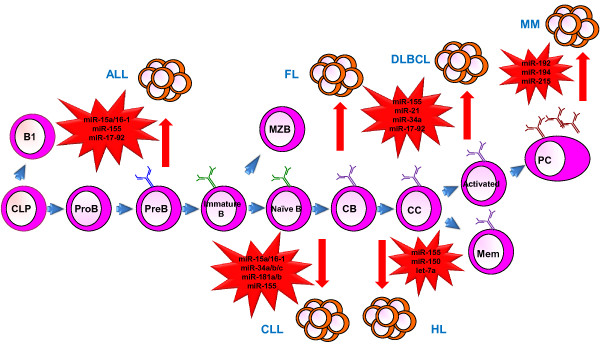
**miRNA dysregulation leads to B cell malignancies**. Dysregulation of key miRNAs at different stages in B cell development can cause malignant transformation and expansion. ALL: B-lymphoblastic lymphoma; CLL: Chronic lymphocytic leukemia; HL: Hodgkin lymphoma; NHL: Non-Hodgkin lymphoma; DLBCL: Diffuse large B cell lymphoma (a type of NHL); FL: Follicular lymphoma (a type of NHL); MM: Multiple Myeloma.

The expression of miRNAs in a particular cell type (miRNome) can vary between normal and diseased tissues. The relationship between miRNAs and cancer was first appreciated when loss of miR-15a/16-1 was discovered in chronic lymphocytic leukemia (CLL) [94]. Also, the discovery that miRNAs were located in cancer-associated genomic regions (CAGRs) furthered studies of the miRNome in a vast number of cancers, from solid tumors to hematological malignancies [95,96]. The function of miRNAs depends greatly on the cellular context for they can act as tumor suppressor genes or oncogenes depending on the genes that are expressed in a given cell. In this section we will focus on miRNAs that have been implicated in B cell lymphoma and leukemia (Figure 2). We will focus our discussion on those miRNAs that have some functional role in B cell development or lymphoma; it is beyond the scope of this review to list the plethora of profiling studies that exist in the literature.

Dysregulation of miR-155 and the miR-15a/16 and miR-17-92 clusters has been implicated in B-ALL [97]. miR-155 overexpression has been observed in certain subtypes of ALL and its over-expression in mice gives rise to B cell lymphoproliferative disease by targeting the SHIP1 inositol phosphatase [98-100]. miR-16 targets anti-apoptotic genes BCL-2, MCL1 and CDK46, thereby acting as a putative tumor suppressor [101]. The miR-17-92 cluster is upregulated in ALL due to copy number amplification and direct upregulation, and transgenic overexpression of this cluster leads to a lymphoproliferative disease in mice, as we have previously discussed (see preceding section on miRNAs and bone marrow B cell development [71,97]). Interestingly, overexpression of a single miRNA, miR-21, can lead to a high-grade B-ALL in mice and similar to protein-coding oncogenes, demonstrates the phenomenon of "oncogene addiction" [102].

In CLL miR-15a/16-1 dysregulation is observed along with dysregulation of miR-34a, miR-34b, miR-34c, miR-181b, miR-181a and miR-155. In fact miR-34a expression has recently been validated as a prognostic marker in CLL in a fairly large clinical study [103,104]. It is interesting to note that one of the most frequent abnormalities in CLL is 13q14 deletion, and the search for candidate tumor suppressor genes in the deleted region had not been successful in the pre-miRNA era [105]. The minimal deleted region (MDR) within 13q14 contains a long non-coding RNA (lncRNA) named DLEU2 [106,107]. Carlo Croce and his colleagues found that the miR-15a/16- cluster was located within intron 4 of DLEU2 [95]. This identification of the first tumor suppressor miRNA was followed by extensive study and delineation of multiple targets [103]. However, the formal assessment of tumor suppressor function was completed much more recently. In an exacting study, Dalla-Favera and colleagues created conditional knockout mice with deletions of the minimal deleted region (MDR), deleting both DLEU2 and the miRNA, or of the miRNA cluster only [108]. Remarkably, the mice with deletion of the miRNA only showed a pre-leukemic expansion of B cells, while the mice with deletion of the MDR developed a CLL-like disease. In this study, the authors showed that there were likely multiple miRNA targets responsible for the phenotypes observed, including some that had been previously identified.

Dysregulation of miRNAs has also been described in Hodgkin's Lymphoma (HL) and Non-Hodgkin's Lymphoma (NHL). In NHL miR-155, miR-21, miR-34a and the miR-17-92 clusters have been implicated. miR-155 carries prognostic implications as high expression of the miRNA is typical of ABC-DLBCL which has a 5 year survival rate [109]. *In vivo *studies demonstrate an oncogenic role for miR-21 in B-lymphomagenesis [102]. Mice expressing miR-21 showed a pre-B malignant lymphoid-like phenotype and inactivating miR-21 caused regression of the malignancy. The miR-17-92 cluster is upregulated in approximately 65% of lymphomas [110]. Furthermore, let-7a, miR-150 and miR-155 are found dysregulated in HL. Let-7a is upregulated causing low expression of PRDM1/Blimp, presumably interrupting plasma cell differentiation [111]. Downregulation of miR-150 and upregulation of miR-155 is common in HL. In normal lymphopoiesis there are high levels of miR-155 in the germinal center, where HL has its origin, suggesting that miR-155 upregulation in HL is due to an abnormal block of lymphocyte differentiation at the germinal center stage [24].

Lastly, p53-mediated miRNA regulation has been found to be important in multiple myeloma (MM), a neoplasm of mature plasma cells. In an effort to understand the miRNA effectors of p53 in this context, Pichiorri and colleagues defined a set of p53-regulated miRNAs, which include miR-192, miR-194, and miR-215 that are downregulated in a subset of newly diagnosed MM. These miRNAs target and downregulate MDM2, a negative regulator of p53. Hence, the expression of these miRNAs reinforces the activity of p53, and the authors found that enforced expression of these miRNAs had a negative effect on the growth of MM cells. Therapeutic possibilities are suggested by the effects of miRNA reconstitution in tempering MM cell growth [112].

The preceding discussion should establish the contribution of miRNAs in lymphoid tumorigenesis. Although some molecular effectors of the miRNnome are known, much remains to be discovered. miRNAs are likely to be of use as diagnostic biomarkers for cancer and as prognostic indicators. Additional work to uncover the roles of miRNAs as therapeutic agents remains to be completed, where a major limitation remains delivery of small RNAs into lymphoid cells.

### MicroRNAs in autoimmunity

Strong responses to self-antigens are thought to be the basis of autoimmune diseases. Many autoimmune diseases are heavily dependent on T cells, but B cells are almost certainly involved, for example, in the secretion of autoantibodies. Indeed mice with a conditional deletion of Dicer in mature B cells develop abnormal B cell subsets, have high autoantibody titers, and female mice develop autoimmune disease with end-organ damage. Several specific miRNAs have been found to be dysregulated in variety of autoimmune disease, and many have a role in T-cell function [113].

It has been found that miR-146a null mice develop a severe autoimmune disorder characterized by enlarged spleen and lymph nodes. These null mice produced about 60 fold higher amounts of autoantibodies against double standard DNA than wild type mice. Autoimmune phenotype in miR-146a null mice is consistent with the finding of elevated amount of activated T cells in the periphery, but may also be dependent on increased activation of B cells [89]. miR-146a also plays a role in the pathogenesis of Systemic lupus erythematosus (SLE). It represses the function of IFN (type one interferon), a factor that is important in SLE, by repressing the target genes such as TRAF6/IRAK1, STAT1 and TLR7 or TLR9 [114-117].

It has been found out that the generation and function of regulatory T-cells (T reg) in autoimmunity is dependent on Dicer dependent miRNA biosynthesis pathway. Mice that have conditional deletion of Dicer in T reg cells showed early onset of autoimmunity which is similar to the observed phenotype in Foxp3 mutant mice that completely lack T reg cells [118,119]. Later study showed that Foxp3 regulate the expression of miR-155 in T reg cells and deficiency of miR-155 results in decreased number, proliferation and fitness of T reg cells compared to wild type [120]. In a similar set of experiments, miR-146a-deficient hematopoietic cells failed to rescue Foxp3-deficient T-cell-mediated autoimmunity [121].

Although the role of miRNAs in B cell-mediated autoimmunity is less firmly established, it is likely that further discoveries in B cells are forthcoming. Finally, it has been shown in a pilot study that miRNA can be potentially used as biomarkers for diagnosis and prognosis of autoimmune diseases such as rheumatic diseases [122].

## Conclusions

The advances in understanding the biological and pathological roles of miRNAs in B cells have been tremendous in the last few years. Despite this progress, there are many questions that remain. The first is how extensive are the networks that are controlled by miRNAs in B cells? Although some studies, including our own, have shown that a single or few targets may be critical at a given developmental stage, it remains to be delineated whether this is generally true or if there are multiple targets that a miRNA regulates. A second major question, which remains largely unexplored in mammalian systems, is how miRNA degradation is regulated. This will help define how gene expression programs may be regulated at one stage but not another by a given miRNA. Lastly, the utilization of miRNA-based therapeutics in B cell malignancies and inflammatory conditions is an area of active research. There are several avenues of promising work that suggests that we will be able to leverage miRNA-based pathways in treating these diseases, but current challenges include delivery into specific cell-types [123]. Research into viral vector-based delivery and into chemically modified small RNA sequences are particularly promising, and are likely to be the next frontiers.

## Competing interests

The authors declare that they have no competing interests.

## Authors' contributions

TF and NIRM participated in its design and coordination and drafted the manuscript. DR conceived of the review, and participated in its design and coordination and drafted the manuscript. All authors read and approved the final manuscript.

## References

[B1] Fire A Xu S Montgomery MK Kostas SA Driver SE Mello CC Potent and specific genetic interference by double-stranded RNA in Caenorhabditis elegans Nature 1998 391 806 811 10.1038/35888 9486653

[B2] Lee RC Feinbaum RL Ambros V The C. elegans heterochronic gene lin-4 encodes small RNAs with antisense complementarity to lin-14 Cell 1993 75 843 854 10.1016/0092-8674(93)90529-Y 8252621

[B3] Ruvkun G Giusto J The Caenorhabditis elegans heterochronic gene lin-14 encodes a nuclear protein that forms a temporal developmental switch Nature 1989 338 313 319 10.1038/338313a0 2922060

[B4] Bernstein E Caudy AA Hammond SM Hannon GJ Role for a bidentate ribonuclease in the initiation step of RNA interference Nature 2001 409 363 366 10.1038/35053110 11201747

[B5] Hammond SM Boettcher S Caudy AA Kobayashi R Hannon GJ Argonaute2, a link between genetic and biochemical analyses of RNAi Science (New York, NY 2001 293 1146 1150 10.1126/science.1064023 11498593

[B6] Ketting RF Fischer SE Bernstein E Sijen T Hannon GJ Plasterk RH Dicer functions in RNA interference and in synthesis of small RNA involved in developmental timing in C. elegans Genes Dev 2001 15 2654 2659 10.1101/gad.927801 11641272 PMC312808

[B7] Ambros V Lee RC Lavanway A Williams PT Jewell D MicroRNAs and other tiny endogenous RNAs in C. elegans Curr Biol 2003 13 807 818 10.1016/S0960-9822(03)00287-2 12747828

[B8] Wightman B Ha I Ruvkun G Posttranscriptional regulation of the heterochronic gene lin-14 by lin-4 mediates temporal pattern formation in C. elegans Cell 1993 75 855 862 10.1016/0092-8674(93)90530-4 8252622

[B9] Kim VN Han J Siomi MC Biogenesis of small RNAs in animals Nat Rev Mol Cell Biol 2009 10 126 139 10.1038/nrm2632 19165215

[B10] Lee Y Kim M Han J Yeom KH Lee S Baek SH Kim VN MicroRNA genes are transcribed by RNA polymerase II EMBO J 2004 23 4051 4060 10.1038/sj.emboj.7600385 15372072 PMC524334

[B11] Lee Y Ahn C Han J Choi H Kim J Yim J Lee J Provost P Radmark O Kim S Kim VN The nuclear RNase III Drosha initiates microRNA processing Nature 2003 425 415 419 10.1038/nature01957 14508493

[B12] Gregory RI Yan KP Amuthan G Chendrimada T Doratotaj B Cooch N Shiekhattar R The Microprocessor complex mediates the genesis of microRNAs Nature 2004 432 235 10.1038/nature03120 15531877

[B13] Lund E Guttinger S Calado A Dahlberg JE Kutay U Nuclear export of microRNA precursors Science (New York, NY 2004 303 95 98 10.1126/science.1090599 14631048

[B14] Bartel DP MicroRNAs: target recognition and regulatory functions Cell 2009 136 215 233 10.1016/j.cell.2009.01.002 19167326 PMC3794896

[B15] Grimson A Farh KK Johnston WK Garrett-Engele P Lim LP Bartel DP MicroRNA Targeting Specificity in Mammals: Determinants beyond Seed Pairing Mol Cell 2007 27 91 105 10.1016/j.molcel.2007.06.017 17612493 PMC3800283

[B16] Lewis BP Burge CB Bartel DP Conserved seed pairing, often flanked by adenosines, indicates that thousands of human genes are microRNA targets Cell 2005 120 15 20 10.1016/j.cell.2004.12.035 15652477

[B17] Brennecke J Stark A Russell RB Cohen SM Principles of microRNA-target recognition PLoS biology 2005 3 e85 10.1371/journal.pbio.0030085 15723116 PMC1043860

[B18] Czech B Hannon GJ Small RNA sorting: matchmaking for Argonautes Nat Rev Genet 2011 12 19 31 21116305 10.1038/nrg2916 PMC3703915

[B19] Cifuentes D Xue H Taylor DW Patnode H Mishima Y Cheloufi S Ma E Mane S Hannon GJ Lawson ND A novel miRNA processing pathway independent of Dicer requires Argonaute2 catalytic activity Science (New York, NY 2010 328 1694 1698 10.1126/science.1190809 PMC3093307 20448148

[B20] Carmell MA Xuan Z Zhang MQ Hannon GJ The Argonaute family: tentacles that reach into RNAi, developmental control, stem cell maintenance, and tumorigenesis Genes Dev 2002 16 2733 2742 10.1101/gad.1026102 12414724

[B21] Sasaki T Shiohama A Minoshima S Shimizu N Identification of eight members of the Argonaute family in the human genome small star, filled Genomics 2003 82 323 330 10.1016/S0888-7543(03)00129-0 12906857

[B22] Xu S Guo K Zeng Q Huo J Lam KP The RNase III enzyme Dicer is essential for germinal center B-cell formation Blood 2012 119 767 776 10.1182/blood-2011-05-355412 22117047

[B23] Belver L de Yebenes VG Ramiro AR MicroRNAs prevent the generation of autoreactive antibodies Immunity 2010 33 713 722 10.1016/j.immuni.2010.11.010 21093320 PMC3687137

[B24] Fabbri M Croce CM Role of microRNAs in lymphoid biology and disease Current Opinion in Hematology 2011 18 266 272 10.1097/MOH.0b013e3283476012 21519241 PMC3400499

[B25] Shaffer AL Lin KI Kuo TC Yu X Hurt EM Rosenwald A Giltnane JM Yang L Zhao H Calame K Staudt LM Blimp-1 orchestrates plasma cell differentiation by extinguishing the mature B cell gene expression program Immunity 2002 17 51 62 10.1016/S1074-7613(02)00335-7 12150891

[B26] Hardy RR Hayakawa K B cell development pathways Annu Rev Immunol 2001 19 595 621 10.1146/annurev.immunol.19.1.595 11244048

[B27] Perez-Vera P Reyes-Leon A Fuentes-Panana EM Signaling proteins and transcription factors in normal and malignant early B cell development Bone marrow research 2011 2011 502751 22046564 10.1155/2011/502751 PMC3200079

[B28] Baltimore D Boldin MP O'Connell RM Rao DS Taganov KD MicroRNAs: new regulators of immune cell development and function Nature immunology 2008 9 839 845 10.1038/ni.f.209 18645592

[B29] Nagasawa T Microenvironmental niches in the bone marrow required for B-cell development Nature reviews Immunology 2006 6 107 116 10.1038/nri1780 16491135

[B30] Arawaka S Wada M Goto S Karube H Sakamoto M Ren CH Koyama S Nagasawa H Kimura H Kawanami T The role of G-protein-coupled receptor kinase 5 in pathogenesis of sporadic Parkinson's disease The Journal of neuroscience: the official journal of the Society for Neuroscience 2006 26 9227 9238 10.1523/JNEUROSCI.0341-06.2006 16957079 PMC6674490

[B31] Nussenweig RS Human trials of malaria vaccine Science (New York, NY 1987 236 763 10.1126/science.3554508 3554508

[B32] Murphy K Janeway's Immunobiology Book Janeway's Immunobiology 2012 8 City: Garland Science, Taylor & Francis Group, LLC

[B33] Nemazee D Buerki K Clonal deletion of autoreactive B lymphocytes in bone marrow chimeras Proc Natl Acad Sci USA 1989 86 8039 8043 10.1073/pnas.86.20.8039 2682636 PMC298209

[B34] Nutt SL Kee BL The transcriptional regulation of B cell lineage commitment Immunity 2007 26 715 725 10.1016/j.immuni.2007.05.010 17582344

[B35] Hu H Wang B Borde M Nardone J Maika S Allred L Tucker PW Rao A Foxp1 is an essential transcriptional regulator of B cell development Nature immunology 2006 7 819 826 10.1038/ni1358 16819554

[B36] Kikuchi K Kondo M Developmental switch of mouse hematopoietic stem cells from fetal to adult type occurs in bone marrow after birth Proc Natl Acad Sci USA 2006 103 17852 17857 10.1073/pnas.0603368103 17090683 PMC1693836

[B37] Yao Z Cui Y Watford WT Bream JH Yamaoka K Hissong BD Li D Durum SK Jiang Q Bhandoola A Stat5a/b are essential for normal lymphoid development and differentiation Proc Natl Acad Sci USA 2006 103 1000 1005 10.1073/pnas.0507350103 16418296 PMC1327727

[B38] Hoelbl A Kovacic B Kerenyi MA Simma O Warsch W Cui Y Beug H Hennighausen L Moriggl R Sexl V Clarifying the role of Stat5 in lymphoid development and Abelson-induced transformation Blood 2006 107 4898 4906 10.1182/blood-2005-09-3596 16493008 PMC2875852

[B39] Bain G Maandag EC Izon DJ Amsen D Kruisbeek AM Weintraub BC Krop I Schlissel MS Feeney AJ van Roon M E2A proteins are required for proper B cell development and initiation of immunoglobulin gene rearrangements Cell 1994 79 885 892 10.1016/0092-8674(94)90077-9 8001125

[B40] Cobaleda C Schebesta A Delogu A Busslinger M Pax5: the guardian of B cell identity and function Nature immunology 2007 8 463 470 10.1038/ni1454 17440452

[B41] Lin YC Jhunjhunwala S Benner C Heinz S Welinder E Mansson R Sigvardsson M Hagman J Espinoza CA Dutkowski J A global network of transcription factors, involving E2A, EBF1 and Foxo1, that orchestrates B cell fate Nature immunology 2010 11 635 643 10.1038/ni.1891 20543837 PMC2896911

[B42] O'Riordan M Grosschedl R Coordinate regulation of B cell differentiation by the transcription factors EBF and E2A Immunity 1999 11 21 31 10.1016/S1074-7613(00)80078-3 10435576

[B43] Rao DS O'Connell RM Chaudhuri AA Garcia-Flores Y Geiger TL Baltimore D MicroRNA-34a perturbs B lymphocyte development by repressing the forkhead box transcription factor Foxp1 Immunity 2010 33 48 59 10.1016/j.immuni.2010.06.013 20598588 PMC2911227

[B44] Look AT Oncogenic transcription factors in the human acute leukemias Science 1997 278 1059 1064 10.1126/science.278.5340.1059 9353180

[B45] Smith E Sigvardsson M The roles of transcription factors in B lymphocyte commitment, development, and transformation J Leukoc Biol 2004 75 973 981 10.1189/jlb.1103554 14982952

[B46] Mullighan CG Goorha S Radtke I Miller CB Coustan-Smith E Dalton JD Girtman K Mathew S Ma J Pounds SB Genome-wide analysis of genetic alterations in acute lymphoblastic leukaemia Nature 2007 446 758 764 10.1038/nature05690 17344859

[B47] Familiades J Bousquet M Lafage-Pochitaloff M Bene MC Beldjord K De Vos J Dastugue N Coyaud E Struski S Quelen C PAX5 mutations occur frequently in adult B-cell progenitor acute lymphoblastic leukemia and PAX5 haploinsufficiency is associated with BCR-ABL1 and TCF3-PBX1 fusion genes: a GRAALL study Leukemia 2009 23 1989 1998 10.1038/leu.2009.135 19587702

[B48] Bhojwani D Howard SC Pui CH High-risk childhood acute lymphoblastic leukemia Clin Lymphoma Myeloma 2009 9 Suppl 3 S222 230 19778845 10.3816/CLM.2009.s.016 PMC2814411

[B49] Mullighan CG Miller CB Radtke I Phillips LA Dalton J Ma J White D Hughes TP Le Beau MM Pui CH BCR-ABL1 lymphoblastic leukaemia is characterized by the deletion of Ikaros Nature 2008 453 110 114 10.1038/nature06866 18408710

[B50] Sun L Heerema N Crotty L Wu X Navara C Vassilev A Sensel M Reaman GH Uckun FM Expression of dominant-negative and mutant isoforms of the antileukemic transcription factor Ikaros in infant acute lymphoblastic leukemia Proc Natl Acad Sci USA 1999 96 680 685 10.1073/pnas.96.2.680 9892693 PMC15196

[B51] Nakase K Ishimaru F Avitahl N Dansako H Matsuo K Fujii K Sezaki N Nakayama H Yano T Fukuda S Dominant negative isoform of the Ikaros gene in patients with adult B-cell acute lymphoblastic leukemia Cancer Res 2000 60 4062 4065 10945610

[B52] Olivero S Maroc C Beillard E Gabert J Nietfeld W Chabannon C Tonnelle C Detection of different Ikaros isoforms in human leukaemias using real-time quantitative polymerase chain reaction Br J Haematol 2000 110 826 830 10.1046/j.1365-2141.2000.02297.x 11054064

[B53] Nishii K Katayama N Miwa H Shikami M Usui E Masuya M Araki H Lorenzo F Ogawa T Kyo T Non-DNA-binding Ikaros isoform gene expressed in adult B-precursor acute lymphoblastic leukemia Leukemia 2002 16 1285 1292 10.1038/sj.leu.2402533 12094252

[B54] Takanashi M Yagi T Imamura T Tabata Y Morimoto A Hibi S Ishii E Imashuku S Expression of the Ikaros gene family in childhood acute lymphoblastic leukaemia Br J Haematol 2002 117 525 530 10.1046/j.1365-2141.2002.03487.x 12028018

[B55] Meleshko AN Movchan LV Belevtsev MV Savitskaja TV Relative expression of different Ikaros isoforms in childhood acute leukemia Blood Cells Mol Dis 2008 41 278 283 10.1016/j.bcmd.2008.06.006 18675565

[B56] Mullighan CG Downing JR Global genomic characterization of acute lymphoblastic leukemia Semin Hematol 2009 46 3 15 10.1053/j.seminhematol.2008.09.005 19100363 PMC2834786

[B57] Georgopoulos K Bigby M Wang JH Molnar A Wu P Winandy S Sharpe A The Ikaros gene is required for the development of all lymphoid lineages Cell 1994 79 143 156 10.1016/0092-8674(94)90407-3 7923373

[B58] Morgan B Sun L Avitahl N Andrikopoulos K Ikeda T Gonzales E Wu P Neben S Georgopoulos K Aiolos, a lymphoid restricted transcription factor that interacts with Ikaros to regulate lymphocyte differentiation EMBO J 1997 16 2004 2013 10.1093/emboj/16.8.2004 9155026 PMC1169803

[B59] Streubel B Vinatzer U Lamprecht A Raderer M Chott A T(3;14)(p14.1;q32) involving IGH and FOXP1 is a novel recurrent chromosomal aberration in MALT lymphoma Leukemia 2005 19 652 658 15703784 10.1038/sj.leu.2403644

[B60] Wlodarska I Veyt E De Paepe P Vandenberghe P Nooijen P Theate I Michaux L Sagaert X Marynen P Hagemeijer A De Wolf-Peeters C FOXP1, a gene highly expressed in a subset of diffuse large B-cell lymphoma, is recurrently targeted by genomic aberrations Leukemia 2005 19 1299 1305 10.1038/sj.leu.2403813 15944719

[B61] Montecino-Rodriguez E Dorshkind K B-1 B cell development in the fetus and adult Immunity 2012 36 13 21 10.1016/j.immuni.2011.11.017 22284417 PMC3269035

[B62] Shapiro-Shelef M Calame K Regulation of plasma-cell development Nature reviews Immunology 2005 5 230 242 10.1038/nri1572 15738953

[B63] Klein U Tu Y Stolovitzky GA Keller JL Haddad J Jr Miljkovic V Cattoretti G Califano A Dalla-Favera R Transcriptional analysis of the B cell germinal center reaction Proc Natl Acad Sci USA 2003 100 2639 2644 10.1073/pnas.0437996100 12604779 PMC151393

[B64] Odegard VH Schatz DG Targeting of somatic hypermutation Nature reviews Immunology 2006 6 573 583 10.1038/nri1896 16868548

[B65] McHeyzer-Williams LJ McHeyzer-Williams MG Antigen-specific memory B cell development Annu Rev Immunol 2005 23 487 513 10.1146/annurev.immunol.23.021704.115732 15771579

[B66] Klein U Casola S Cattoretti G Shen Q Lia M Mo T Ludwig T Rajewsky K Dalla-Favera R Transcription factor IRF4 controls plasma cell differentiation and class-switch recombination Nature immunology 2006 7 773 782 10.1038/ni1357 16767092

[B67] Cambier JC Gauld SB Merrell KT Vilen BJ B-cell anergy: from transgenic models to naturally occurring anergic B cells? Nat Rev Immunol 2007 7 633 643 10.1038/nri2133 17641666 PMC3714009

[B68] Bartel DP Chen CZ Micromanagers of gene expression: the potentially widespread influence of metazoan microRNAs Nat Rev Genet 2004 5 396 400 15143321 10.1038/nrg1328

[B69] Chen CZ Li L Lodish HF Bartel DP MicroRNAs modulate hematopoietic lineage differentiation Science (New York, NY 2004 303 83 86 10.1126/science.1091903 14657504

[B70] Koralov SB Muljo SA Galler GR Krek A Chakraborty T Kanellopoulou C Jensen K Cobb BS Merkenschlager M Rajewsky N Rajewsky K Dicer ablation affects antibody diversity and cell survival in the B lymphocyte lineage Cell 2008 132 860 874 10.1016/j.cell.2008.02.020 18329371

[B71] Xiao C Srinivasan L Calado DP Patterson HC Zhang B Wang J Henderson JM Kutok JL Rajewsky K Lymphoproliferative disease and autoimmunity in mice with increased miR-17-92 expression in lymphocytes Nature immunology 2008 9 405 414 10.1038/ni1575 18327259 PMC2533767

[B72] Xiao C Calado DP Galler G Thai TH Patterson HC Wang J Rajewsky N Bender TP Rajewsky K MiR-150 controls B cell differentiation by targeting the transcription factor c-Myb Cell 2007 131 146 159 10.1016/j.cell.2007.07.021 17923094

[B73] Zhou B Wang S Mayr C Bartel DP Lodish HF miR-150, a microRNA expressed in mature B and T cells, blocks early B cell development when expressed prematurely Proc Natl Acad Sci USA 2007 104 7080 7085 10.1073/pnas.0702409104 17438277 PMC1855395

[B74] Xiao C Rajewsky K MicroRNA control in the immune system: basic principles Cell 2009 136 26 36 10.1016/j.cell.2008.12.027 19135886

[B75] Meffre E Casellas R Nussenzweig MC Antibody regulation of B cell development Nature immunology 2000 1 379 385 10.1038/80816 11062496

[B76] He L He X Lim LP de Stanchina E Xuan Z Liang Y Xue W Zender L Magnus J Ridzon D A microRNA component of the p53 tumour suppressor network Nature 2007 447 1130 1134 10.1038/nature05939 17554337 PMC4590999

[B77] Chang T-C Wentzel EA Kent OA Ramachandran K Mullendore M Lee KwangÂ H Feldmann G Yamakuchi M Ferlito M Lowenstein CJ Transactivation of miR-34a by p53 Broadly Influences Gene Expression and Promotes Apoptosis Molecular Cell 2007 26 745 752 10.1016/j.molcel.2007.05.010 17540599 PMC1939978

[B78] Bommer GT Gerin I Feng Y Kaczorowski AJ Kuick R Love RE Zhai Y Giordano TJ Qin ZS Moore BB p53-Mediated Activation of miRNA34 Candidate Tumor-Suppressor Genes Curr Biol 2007 17 1298 1307 10.1016/j.cub.2007.06.068 17656095

[B79] O'Connell RM Chaudhuri AA Rao DS Gibson WS Balazs AB Baltimore D MicroRNAs enriched in hematopoietic stem cells differentially regulate long-term hematopoietic output Proc Natl Acad Sci USA 2010 107 14235 14240 10.1073/pnas.1009798107 20660734 PMC2922591

[B80] Georgantas RW Hildreth R Morisot S Alder J Liu CG Heimfeld S Calin GA Croce CM Civin CI CD34+ hematopoietic stem-progenitor cell microRNA expression and function: a circuit diagram of differentiation control Proc Natl Acad Sci USA 2007 104 2750 2755 10.1073/pnas.0610983104 17293455 PMC1796783

[B81] Gibcus JH Tan LP Harms G Schakel RN de Jong D Blokzijl T Moller P Poppema S Kroesen BJ van den Berg A Hodgkin lymphoma cell lines are characterized by a specific miRNA expression profile Neoplasia (New York, NY 2009 11 167 176 10.1593/neo.08980 PMC2631141 19177201

[B82] O'Connell RM Taganov KD Boldin MP Cheng G Baltimore D MicroRNA-155 is induced during the macrophage inflammatory response Proc Natl Acad Sci USA 2007 104 1604 1609 10.1073/pnas.0610731104 17242365 PMC1780072

[B83] Taganov KD Boldin MP Chang KJ Baltimore D NF-kappaB-dependent induction of microRNA miR-146, an inhibitor targeted to signaling proteins of innate immune responses Proc Natl Acad Sci USA 2006 103 12481 12486 10.1073/pnas.0605298103 16885212 PMC1567904

[B84] Rodriguez A Vigorito E Clare S Warren MV Couttet P Soond DR van Dongen S Grocock RJ Das PP Miska EA Requirement of bic/microRNA-155 for normal immune function Science (New York, NY 2007 316 608 611 10.1126/science.1139253 PMC2610435 17463290

[B85] Vigorito E Perks KL Abreu-Goodger C Bunting S Xiang Z Kohlhaas S Das PP Miska EA Rodriguez A Bradley A microRNA-155 regulates the generation of immunoglobulin class-switched plasma cells Immunity 2007 27 847 859 10.1016/j.immuni.2007.10.009 18055230 PMC4135426

[B86] Teng G Hakimpour P Landgraf P Rice A Tuschl T Casellas R Papavasiliou FN MicroRNA-155 is a negative regulator of activation-induced cytidine deaminase Immunity 2008 28 621 629 10.1016/j.immuni.2008.03.015 18450484 PMC2430982

[B87] Dorsett Y McBride KM Jankovic M Gazumyan A Thai TH Robbiani DF Di Virgilio M San-Martin BR Heidkamp G Schwickert TA MicroRNA-155 suppresses activation-induced cytidine deaminase-mediated Myc-Igh translocation Immunity 2008 28 630 638 10.1016/j.immuni.2008.04.002 18455451 PMC2713656

[B88] de Yebenes VG Belver L Pisano DG Gonzalez S Villasante A Croce C He L Ramiro AR miR-181b negatively regulates activation-induced cytidine deaminase in B cells The Journal of Experimental Medicine 2008 205 2199 2206 10.1084/jem.20080579 18762567 PMC2556787

[B89] Boldin MP Taganov KD Rao DS Yang L Zhao JL Kalwani M Garcia-Flores Y Luong M Devrekanli A Xu J miR-146a is a significant brake on autoimmunity, myeloproliferation, and cancer in mice The Journal of Experimental Medicine 2011 208 1189 1201 10.1084/jem.20101823 21555486 PMC3173243

[B90] Cameron JE Yin Q Fewell C Lacey M McBride J Wang X Lin Z Schaefer BC Flemington EK Epstein-Barr virus latent membrane protein 1 induces cellular MicroRNA miR-146a, a modulator of lymphocyte signaling pathways J Virol 2008 82 1946 1958 10.1128/JVI.02136-07 18057241 PMC2258704

[B91] Kawai T Akira S Signaling to NF-kappaB by Toll-like receptors Trends Mol Med 2007 13 460 469 10.1016/j.molmed.2007.09.002 18029230

[B92] Zhao JL Rao DS Boldin MP Taganov KD O'Connell RM Baltimore D NF-kappaB dysregulation in microRNA-146a-deficient mice drives the development of myeloid malignancies Proc Natl Acad Sci USA 2011 108 9184 9189 10.1073/pnas.1105398108 21576471 PMC3107319

[B93] Gururajan M Haga CL Das S Leu CM Hodson D Josson S Turner M Cooper MD MicroRNA 125b inhibition of B cell differentiation in germinal centers Int Immunol 2010 22 583 592 10.1093/intimm/dxq042 20497960 PMC2892362

[B94] Calin GA Ferracin M Cimmino A Di Leva G Shimizu M Wojcik SE Iorio MV Visone R Sever NI Fabbri M A MicroRNA Signature Associated with Prognosis and Progression in Chronic Lymphocytic Leukemia The New England Journal of Medicine 2005 353 1793 1801 10.1056/NEJMoa050995 16251535

[B95] Calin GA Dumitru CD Shimizu M Bichi R Zupo S Noch E Aldler H Rattan S Keating M Rai K Frequent deletions and down-regulation of micro- RNA genes miR15 and miR16 at 13q14 in chronic lymphocytic leukemia Proc Natl Acad Sci USA 2002 99 15524 15529 10.1073/pnas.242606799 12434020 PMC137750

[B96] Calin GA Sevignani C Dumitru CD Hyslop T Noch E Yendamuri S Shimizu M Rattan S Bullrich F Negrini M Croce CM Human microRNA genes are frequently located at fragile sites and genomic regions involved in cancers Proceedings of the National Academy of Sciences of the United States of America 2004 101 2999 3004 10.1073/pnas.0307323101 14973191 PMC365734

[B97] Mi S Li Z Chen P He C Cao D Elkahloun A Lu J Pelloso LA Wunderlich M Huang H Aberrant overexpression and function of the miR-17-92 cluster in MLL-rearranged acute leukemia Proc Natl Acad Sci USA 2010 107 3710 3715 10.1073/pnas.0914900107 20133587 PMC2840429

[B98] Costinean S Zanesi N Pekarsky Y Tili E Volinia S Heerema N Croce CM Pre-B cell proliferation and lymphoblastic leukemia/high-grade lymphoma in E(mu)-miR155 transgenic mice Proc Natl Acad Sci USA 2006 103 7024 7029 10.1073/pnas.0602266103 16641092 PMC1459012

[B99] 99. Costinean S Sandhu SK Pedersen IM Tili E Trotta R Perrotti D Ciarlariello D Neviani P Harb J Kauffman LR Src homology 2 domain-containing inositol-5-phosphatase and CCAAT enhancer-binding protein beta are targeted by miR-155 in B cells of Emicro-MiR-155 transgenic mice Blood 2009 114 1374 1382 10.1182/blood-2009-05-220814 19520806 PMC2727407

[B100] Garzon R Volinia S Liu CG Fernandez-Cymering C Palumbo T Pichiorri F Fabbri M Coombes K Alder H Nakamura T MicroRNA signatures associated with cytogenetics and prognosis in acute myeloid leukemia Blood 2008 111 3183 3189 10.1182/blood-2007-07-098749 18187662 PMC2265455

[B101] Cimmino A Calin GA Fabbri M Iorio MV Ferracin M Shimizu M Wojcik SE Aqeilan RI Zupo S Dono M miR-15 and miR-16 induce apoptosis by targeting BCL2 Proc Natl Acad Sci USA 2005 102 13944 13949 10.1073/pnas.0506654102 16166262 PMC1236577

[B102] Medina PP Nolde M Slack FJ OncomiR addiction in an in vivo model of microRNA-21-induced pre-B-cell lymphoma Nature 2010 467 86 90 10.1038/nature09284 20693987

[B103] Fabbri M Bottoni A Shimizu M Spizzo R Nicoloso MS Rossi S Barbarotto E Cimmino A Adair B Wojcik SE Association of a microRNA/TP53 feedback circuitry with pathogenesis and outcome of B-cell chronic lymphocytic leukemia JAMA 2011 305 59 67 10.1001/jama.2010.1919 21205967 PMC3690301

[B104] Zenz T Mohr J Eldering E Kater AP Buhler A Kienle D Winkler D Durig J van Oers MH Mertens D miR-34a as part of the resistance network in chronic lymphocytic leukemia Blood 2009 113 3801 3808 10.1182/blood-2008-08-172254 18941118

[B105] Chiorazzi N Rai KR Ferrarini M Chronic lymphocytic leukemia N Engl J Med 2005 352 804 815 10.1056/NEJMra041720 15728813

[B106] Liu Y Corcoran M Rasool O Ivanova G Ibbotson R Grander D Iyengar A Baranova A Kashuba V Merup M Cloning of two candidate tumor suppressor genes within a 10 kb region on chromosome 13q14, frequently deleted in chronic lymphocytic leukemia Oncogene 1997 15 2463 2473 10.1038/sj.onc.1201643 9395242

[B107] 107. Migliazza A Bosch F Komatsu H Cayanis E Martinotti S Toniato E Guccione E Qu X Chien M Murty VV Nucleotide sequence, transcription map, and mutation analysis of the 13q14 chromosomal region deleted in B-cell chronic lymphocytic leukemia Blood 2001 97 2098 2104 10.1182/blood.V97.7.2098 11264177

[B108] Klein U Lia M Crespo M Siegel R Shen Q Mo T Ambesi-Impiombato A Califano A Migliazza A Bhagat G Dalla-Favera R The DLEU2/miR-15a/16-1 cluster controls B cell proliferation and its deletion leads to chronic lymphocytic leukemia Cancer Cell 2010 17 28 40 10.1016/j.ccr.2009.11.019 20060366

[B109] Lawrie CH Soneji S Marafioti T Cooper CD Palazzo S Paterson JC Cattan H Enver T Mager R Boultwood J MicroRNA expression distinguishes between germinal center B cell-like and activated B cell-like subtypes of diffuse large B cell lymphoma International Journal of Cancer 2007 121 1156 1161 10.1002/ijc.22800 17487835

[B110] Ota A Tagawa H Karnan S Tsuzuki S Karpas A Kira S Yoshida Y Seto M Identification and characterization of a novel gene, C13orf25, as a target for 13q31-q32 amplification in malignant lymphoma Cancer Res 2004 64 3087 3095 10.1158/0008-5472.CAN-03-3773 15126345

[B111] 111. Nie K Gomez M Landgraf P Garcia JF Liu Y Tan LH Chadburn A Tuschl T Knowles DM Tam W MicroRNA-mediated down-regulation of PRDM1/Blimp-1 in Hodgkin/Reed-Sternberg cells: a potential pathogenetic lesion in Hodgkin lymphomas The American Journal of Pathology 2008 173 242 252 10.2353/ajpath.2008.080009 18583325 PMC2438301

[B112] 112. Pichiorri F Suh SS Rocci A De Luca L Taccioli C Santhanam R Zhou W Benson DM Jr Hofmainster C Alder H Downregulation of p53-inducible microRNAs 192, 194, and 215 impairs the p53/MDM2 autoregulatory loop in multiple myeloma development Cancer Cell 2010 18 367 381 10.1016/j.ccr.2010.09.005 20951946 PMC3561766

[B113] Iborra M Bernuzzi F Invernizzi P Danese S MicroRNAs in autoimmunity and inflammatory bowel disease: Crucial regulators in immune response Autoimmunity reviews 2010 11 305 314 20627134 10.1016/j.autrev.2010.07.002

[B114] Ronnblom L Eloranta ML Alm GV The type I interferon system in systemic lupus erythematosus Arthritis and rheumatism 2006 54 408 420 10.1002/art.21571 16447217

[B115] Pascual V Farkas L Banchereau J Systemic lupus erythematosus: all roads lead to type I interferons Curr Opin Immunol 2006 18 676 682 10.1016/j.coi.2006.09.014 17011763

[B116] Crow MK Type I interferon in systemic lupus erythematosus Current topics in microbiology and immunology 2007 316 359 386 10.1007/978-3-540-71329-6_17 17969456

[B117] Tang Y Luo X Cui H Ni X Yuan M Guo Y Huang X Zhou H de Vries N Tak PP MicroRNA-146A contributes to abnormal activation of the type I interferon pathway in human lupus by targeting the key signaling proteins Arthritis and rheumatism 2009 60 1065 1075 10.1002/art.24436 19333922

[B118] Rudensky AY Regulatory T cells and Foxp3 Immunol Rev 2011 241 260 268 10.1111/j.1600-065X.2011.01018.x 21488902 PMC3077798

[B119] Liston A Lu LF O'Carroll D Tarakhovsky A Rudensky AY Dicer-dependent microRNA pathway safeguards regulatory T cell function The Journal of experimental medicine 2008 205 1993 2004 10.1084/jem.20081062 18725526 PMC2526195

[B120] Lu LF Thai TH Calado DP Chaudhry A Kubo M Tanaka K Loeb GB Lee H Yoshimura A Rajewsky K Rudensky AY Foxp3-dependent microRNA155 confers competitive fitness to regulatory T cells by targeting SOCS1 protein Immunity 2009 30 80 91 10.1016/j.immuni.2008.11.010 19144316 PMC2654249

[B121] Lu LF Boldin MP Chaudhry A Lin LL Taganov KD Hanada T Yoshimura A Baltimore D Rudensky AY Function of miR-146a in controlling Treg cell-mediated regulation of Th1 responses Cell 2010 142 914 929 10.1016/j.cell.2010.08.012 20850013 PMC3049116

[B122] Alevizos I Illei GG MicroRNAs as biomarkers in rheumatic diseases Nature reviews Rheumatology 2010 6 391 398 10.1038/nrrheum.2010.81 20517293 PMC3041596

[B123] Krutzfeldt J Rajewsky N Braich R Rajeev KG Tuschl T Manoharan M Stoffel M Silencing of microRNAs in vivo with 'antagomirs' Nature 2005 438 685 689 10.1038/nature04303 16258535

